# Evolution of the search for a common mechanism of congenital risk of coronary heart disease and type 2 diabetes mellitus in the chromosomal locus 9p21.3

**DOI:** 10.1097/MD.0000000000035074

**Published:** 2023-10-13

**Authors:** Valeriy Benberin, Raushan Karabaeva, Nazgul Kulmyrzaeva, Rauza Bigarinova, Tamara Vochshenkova

**Affiliations:** a Centre of Gerontology, Medical Center Hospital of the President’s Affairs Administration of the Republic of Kazakhstan, Astana, Kazakhstan.

**Keywords:** 9p21.3 chromosomal locus, coronary heart disease, genome-wide association studies (GWAS), single nucleotide polymorphisms (SNPs), type 2 diabetes mellitus 2

## Abstract

9.21.3 chromosomal locus predisposes to coronary heart disease (CHD) and type 2 diabetes mellitus (DM2), but their overall pathological mechanism and clinical applicability remain unclear. The review uses publications of the study results of 9.21.3 chromosomal locus in association with CHD and DM2, which are important for changing the focus of clinical practice. The eligibility criteria are full-text articles published in the PubMed database (MEDLINE) up to December 31, 2022. A total of 56 publications were found that met the inclusion criteria. Using the examples of the progressive stages in understanding the role of the chromosomal locus 9p.21.3, scientific ideas were grouped, from a fragmentary study of independent pathological processes to a systematic study of the overall development of CHD and DM2. The presented review can become a source of new scientific hypotheses for further studies, the results of which can determine the general mechanism of the congenital risk of CHD and DM2 and change the focus of clinical practice.

## 1. Introduction

Almost 75% of patients with coronary heart disease (CHD) have concomitant type 2 diabetes mellitus (DM2) or severe glucose homeostasis disorders, and the number of patients with CHD is gradually rising over the recent years.^[1,2]^ СHD is the main cause of more than 50% of deaths among patients with DM2, and, notably, Asian patients have a higher risk of death than European patients.^[3]^ Additionally, CHD is becoming an increasingly significant cause of disability and mortality among diabetics, reducing their lifespan by ten years.^[3]^

Researchers have observed new epidemiological trends in the prevalence of CHD in individuals with DM2.^[4]^ For instance, since 2000, there has been an increase in the number of clinical complications associated with DM2 in young and middle-aged individuals, including myocardial infarction and stroke. In Hong Kong, Wu X et al have reported a similar trend based on a 16-year observation of the development of DM2 in 770,780 patients aged 20 years and older.^[5]^ Moreover, the active and joint increase of CHD and DM2 was manifested in vascular events in patients who had no prior signs of coronary artery obstruction, making this trend a new challenge for global public health.^[6–9]^

There is a large amount of evidence indicating a genetic risk for the development of CHD and DM2. In particular, the group of observations by Yun JS et al demonstrated that a high genetic risk of CHD or DM2 (about 1% of the population) increased the risk of cardiovascular death by 1.6 and 2.1 times, respectively, versus participants with low genetic risk.^[10]^ The chromosomal locus 9p.21.3 is the second main reason for predisposition for CHD development and the main genetic inducer for DM2 development. However, it is not associated with any of the side effects. It is also worth noting that the positive lifestyle changes to reduce the mortality rate associated with cardiovascular diseases in patients with high polygenic risk of CHD or DM2 seem to be inefficient with only more than 30% success rate, in the absence of an intensive long-term intervention in lifestyle over usual treatments.^[11]^ It has also been reported that genetically predisposed subgroups for DM2 have similar consequences of cardiovascular and cardiometabolic risks.^[12]^ All these recent findings support the hypothesis of the significance of the chromosomal locus in the development of these 2 conditions. Therefore, the growing global burden of CHD and DM2 calls for effective interventions. Clinical and genetic factors are of great importance in the strategy for preventing CHD and DM2, providing valuable information for clinical practice. This article presents a review of clinically relevant publications to understand the role of the chromosomal locus 9p21.3 in the development of DM2 and CHD.

## 2. Materials and methods

### 2.1. The source selection

The review uses clinically relevant publications reflecting the current trends in scientific research on 9p21.3 chromosomal locus in association with CHD and DM2 that were obtained from the PubMed database (MEDLINE) up to December 31, 2022. A total of 86 sources were found, of which 56 were selected that met the inclusion criteria.

### 2.2. Methods of assessment and synthesis

The study was conducted through a sequential comprehensive and systematic literature search. The chromosomal locus 9p21.3 was used as the genetic risk locus of interest, and the outcomes of interest were CHD and DM2.

A literature search of relevant published articles in the PubMed databases (MEDLINE) was performed using the search query (“locus 9p21.3, CHD, DM2”). The most recent search update was on December 31, 2022. Additionally, relevant publications were obtained by searching for references cited in published articles. Article types were limited to reviews, systematic reviews, and original studies. To provide sufficient depth of review, publications included in vitro and in vivo studies.

### 2.3. Ethics

The study is an analytical processing of scientific research materials published in the public database PubMed (MEDLINE), therefore, it is not subject to ethical review.

## 3. Results

The obtained results were considered in terms of the presence of progress and, therefore, the prospects of new knowledge about the investigated locus for clinical practice.

The search for genes involved in monogenic disorders in CHD and DM2 began in the 1990s with the use of large family lineage, followed by an analysis of candidate genes. At that time, formal genetics operated with the abstract concept of genes, linearly arranged in linkage groups, directly or indirectly affecting the manifestation of a trait. Consequently, it led to the observations of the relationship between CHD and DM2 with genetic variants, - in particular, the role of haploids located on chromosomes 11 and 3, for CHD and DM2, respectively, was revealed.^[13,14]^ Genetic relation between early CHD and signs of metabolic syndrome (hyperlipidemia, arterial hypertension, DM2) with a short segment of chromosome 12 has also been identified.^[15]^

In the 2000s, the development of genome-wide association studies (GWAS) using DNA microarrays gave rise to structural genomics. Based on their results, 3 papers were published in 2007 identifying the chromosomal locus 9p21.3 as the strongest genetic signal for CHD in individuals of European descent.^[16–18]^ At the same time, researchers of the genetic risk of DM2 came to approximately the same results.^[19–21]^

The first mention of the locus in connection with both diseases was noted in the publication of a study conducted by the Wellcome Trust Case Control Consortium. The study examined 2000 individuals of the British population for 7 diseases. As a result, it has been identified that some loci are associated with risks for more than one of these diseases.^[22]^ An indirect confirmation of this conclusion was the discovery of 3 single nucleotide polymorphisms (SNPs) located in 2 neighboring haplotypes in the 9p21.3 region, which was associated with DM2 and CHD in the Han Chinese population.^[23]^ Two subsequent studies suggested for the first time that common risk factors for CHD and DM2 do not necessarily imply the presence of common genetic risk factors.^[24,25]^ Thus, new avenues for investigating the pathophysiology of complex disorders have been presented.

The risk haplotypes of the chromosomal locus 9p21.3 are unique in each human individual and do not have coding genes, making it difficult to attempt to decipher its function using GWAS. The possibilities of whole genome and whole exome sequencing have expanded the understanding of the genetic architecture and functional significance of CHD and DM2. It was found that 2 risk haplotypes of these diseases in the locus represent blocks of 50 to 100 SNPs in non-equilibrium linkage, providing nonrandom joint inheritance up to 50% of cases in representatives of many populations.^[26]^ Their prevalence and strong additive effect lead to at least 15% of CHD and DM2 cases, representing the chromosomal locus 9p21.3 as the largest known genomic source of CHD and DM2 development.^[27]^

The first scientific data on the p15/CDKN2B-p16/CDKN2A-p14/gene encoding protein ADP ribosylation factor locus gene cluster were associated with the largest family of 7 syndromes of the melanoma-neural system and detection using heterozygosity mapping based on microsatellite markers of the 9p21 large germline deletion. The results of the study suggested the presence of coordinated regulation of transcription by antisense gene, non-coding RNA, non-coding gene (ANRIL).^[28]^

It should be noted that the data on ANRIL are inconsistent, probably due to the presence of numerous linear and circular isoforms in the gene capable of transformation by back splicing. ANRIL can both positively and negatively regulate the work of genes while acting both locally and remotely. In general, the role of ANRIL is determined by the balance between the level of expression of linear and circular variants; and the molecular differences between variants can lead to fundamental changes in function cells, i.e. the rate of proliferation in smooth muscle cells, pancreatic β-cells, or macrophages. The expression of linear ANRIL is associated with the development of CHD and DM2. Circular ANRIL is recognized as useful for the regulation of homeostasis, it has a higher expression level and is more stable. Even a slight predominance of the linear variant over the circular one will contribute to the development of CHD and DM2.^[8,29]^ Its expression is also influenced by pathological changes in preexisting diseases. The regulation of the balance depends on the cell type and context, however, there is still no sufficient data on the molecular coordinator of the ratio of linear and circular isoforms, and it is not known whether inhibition of linear ANRIL isoforms is sufficient to protect against CHD and DM2.^[30]^ Perhaps this special influence of ANRIL can also explain the paradox when the genes involved in the biology of traits show different effects. Genetic variants can disrupt ANRIL, which mechanically links CHD to DM2.^[31]^ Subsequently, it is hypothesized that 9p21.3 chromosomal locus controls large gene networks that predispose to the adoption of cellular conditions that lead to CHD and DM2.^[32,33]^

Transcriptional changes suggest a broad context of gene interaction and regulation. The integration of DNA and RNA sequencing data that interact in the supernetwork of cardiometabolic diseases within the framework of a system analysis made it possible to form an understanding of the modules of genetic regulation of gene expression. In particular, the relationship between СHD and DM2 at the interorgan level is regulated by a module that binds 553 genes, among the driver-gene loci of which the influence of 9p21.3 was noted.^[34]^ It turned out that the true transmission of endocrine signals is carried from organ to organ by inter-tissue pathways, which determine the specificity of the source and the target. In this case, the phenotype of the target tissue cell acquires prognostic significance in the development of СHD and DM2.^[35]^ The study group of Indumathi B et al, seeking to identify epigenetic signatures characteristic of CHD, concluded that hypomethylation of the locus causes activation of CDKN2A increasing the risk of CHD by 1.79 times, which is on par with similar epigenetic traits that were noted in the early onset of DM2.^[36]^

Thibaut M et al noted the involvement of rs1333049 in the early severe myocardial infarction in the Slovenian population while revealing a positive connection in the control group with individuals with DM2 but without CHD. The propensity for plaque rupture was explained by the low number of smooth muscle cells due to the high expression of CDKN2B-AS1, which revealed the need to use rs1333049 as a genetic marker to isolate a group of patients with DM2 for careful cardiac monitoring.^[37]^ It was previously determined that CHD and myocardial infarction belong to different risk haplotypes at the chromosomal locus 9p21.3.^[24]^ Hence, transcriptional dysregulation as one of the causes for the onset of at least 15% of cases of T2D and ischemic heart disease (IHD) appears compelling.

The traditional reductionist approach prevails in most studies focusing on CHD and DM2. An alternative for representing the complex interaction between the molecular, biological, biochemical, and cellular structures of CHD and DM2 within a single architecture is network medicine. It aims to provide more accurate genotyping of multiple interactions using integrated multi-omics as a comprehensive view.^[38]^ Due to that, it was possible to determine the functions of different participants in the transcription process. A promoter is a sequence of DNA nucleotides recognized by RNA as a launching pad for the start of transcription. The sequence and architecture of the DNA promoter determine the variability in gene expression, representing a selective trade-off between its stability and signaling plasticity. Enhancers activate transcription induced by a particular promoter.^[39]^ The ability of a number of enhancers to interact with specific proteins (transcription factors) in differentiated cells ensures the tissue-specific nature of gene expression. Changes in the sequence of cell type-specific enhancers make a significant contribution to the phenotypic variability of the human population. There are hundreds of different types of cells in the body, but they all have the same genes. Enhancers, acting as a genetic switch, determine which gene needs to be turned on and assemble a short sequence. And transcription factors, specific proteins of the cell, recognize this sequence and begin to work together, contributing to the activation of the gene.^[39]^ The mechanism by which the chromosomal locus 9p21.3 confers increased susceptibility to CHD and DM2 remains unclear. SNPs are likely to disrupt specific regulatory sequences within tissue-specific ANRIL enhancers. The identification of these SNPs and the cells in which they function is of scientific interest for CHD and DM2.

An integrated multi-omics approach that combines transcriptomic and epigenetic data with the data from the cellular phenotypes of the disease is already yielding promising results. The convergence of knowledge and methods in the field of molecular biology, genetics and genomics has made it technically possible and in demand to edit certain base pairs or DNA segments in cells and living organisms. In particular, based on clustered genome editing (CRISPR) technology applicable to human beta-cells, a genomic screening technology was developed and implemented in a human beta cell model, linking the direction of epigenetic activity of the causative genes of the DM2 locus GWAS with the tissue of influence and functional mechanisms of DM2. It can be assumed that such studies using the general cell biology of CHD and DM2 at the level of the chromosomal locus 9p21.3 can also become a source of unexpected discoveries.^[40]^

The increasingly complex approaches to classifying subtypes of heterogeneous diseases require a more precise definition of the underlying biological mechanisms.^[41]^ Since 2017, the increasing amount of publications indicated the existence of membrane-less organelles in every human cell - biomolecular protein condensates. It is assumed that their activities are affected by more than 36,000 pathogenic mutations that contribute to the emergence of almost 1700 diseases.^[42]^ In particular, aberrative protein condensation of metabolic enzymes is associated with DM2 and CHD.^[43]^ To date, there are numerous fairly well-founded objections to this new complex and poorly defined field of scientific research. Nonetheless, biotech companies see condensates as a target for exposure, such as the dissolution of toxic cancer and neurodegenerative disease-inducing proteins in the condensate. The biological pathways that connect condensates with the chromosomal locus 9p21.3 would be the new avenue for the exploration for researchers. Therefore, progress in understanding the biological processes involved in IHD and T2D deepens our understanding of the biological role of the 9p21.3 chromosomal locus and may serve as a source for effective interventions in clinical practice (Table 1).

**Table 1:** Key stages of scientific knowledge development regarding the chromosomal locus 9p21.3 in association with coronary heart disease (CHD) and type 2 diabetes mellitus (DM2).

Stages	Progress in knowledge	The changes for practical medicine
Formal genetics	Understanding the association of IHD and T2D with genetic variants	The presence of a genetic risk for the development of IHD and T2D
Structural genomics	• Identification of the chromosomal locus 9p21.3 in association with IHD and T2D, independent of the identification of disease risk factors. • Identification of rare mutations in the 9p21.3 chromosomal locus as predictors of risk.	• The hypothesis is that the locus represents a previously unknown pathogenic mechanism shared by IHD and T2D. • The synthesis of gene complexes and factors that play a causal role in the development of IHD and T2D.
Functional genomics	Transcriptional regulation	• Transcriptional dysregulation in the 9p21.3 chromosomal locus as a common basis for IHD and T2D. • Expansion of the list of functional mutations in promoters and enhancers.
Genome editing	High-throughput genome screening	Immunometabolism in the context of the common onset of IHD and T2D
Intracellular biology (Protein folding)	Intracellular accumulation of metabolic enzymes in IHD and T2D	New opportunities for personalized medicine: removal of misfolded proteins from cells, drug delivery into cells, etc.

## 4. Discussion

There are 3 possible scenarios for the involvement of the chromosomal locus 9p21.3 in T2D and IHD: (1) each of these conditions has a unique starting point and development; (2) shared development of these 2 conditions but with independent starting points; (3) a common starting point for both conditions with independent subsequent development. Accumulated results support the last scenario based on the following data.

DM2 and CHD are components of a multifaceted disorder at the level of immune metabolism. The key biological processes in humans as multicellular organisms are the targeted production of nutrients in cells and tissues (metabolism) and defense against external infections and damage (immunity). The interconnection processes inevitably involve the metabolism of lipids and glucose, which activate the reprogramming of immune cells. Thus, metabolic signaling governs the functions of cells. The influence of a common immune-metabolic starting point on the development and progression of many diseases, including DM2 and CHD, is highly probable.^[44,45]^

Aberrations in the transcription of a specific DNA sequence in the locus trigger a unified mechanism for the development of both diseases. The fact that immune cells adopt metabolic programs specific to their state and the surrounding environment can serve as an example of the interconnection between metabolic pathways, signaling, and cell differentiation. Identifying the mechanism of transcriptional regulation in the genetic locus that induces inflammatory signaling and tissue proliferation changes allows it to be identified as a common genetic signature for DM2 and CHD, in conjunction with sha red environmental risks and clinical association.^[46]^

Transcriptional regulation is influenced by ANRIL, the product of the CDKN2B-AS gene. The genetic risk associated with cell cycle regulation depends not on the presence of significant risk factors for both diseases but on earlier events. Specifically, the CDKN2B-AS gene, through ANRIL, influences the cellular growth and proliferation of pancreatic beta cells and smooth muscle cells of the vascular wall, forming the future phenotype of “DM2 and CHD.”^[47]^ The locus induced and controlled by ANRIL also contributes to the involvement of various factors at multiple levels of the system, contributing to the diversity of disease endophenotypes unrelated to the 9p21.3 chromosomal locus.

The sequence and architecture of nucleotides in the promoter and enhancer provide the influence of ANRIL on the transmission of inflammatory signals and tissue proliferation changes by altering the expression of genes associated with DM2 and CHD. The influence of the ANRIL transcription gene encompasses a cluster of enhancers, each of which can act depending on the tissue type and exhibit pronounced specificity.

The ability of a series of enhancers to interact with specific proteins in differentiated cells ensures tissue-specific gene expression. The ability of tissues to undergo regeneration is a fundamental driving force against aging—ann innate cellular response controlled, in part, at the 9p21.3 locus. There is a suggestion that in DM2 and CHD, the transcriptional regulation of the locus is primarily mediated by distal regulatory elements.^[48]^ By targeting transcription factors or epigenetic modifiers involved in gene expression regulation, a wide range of effects beyond the direct study of the 9p21.3 locus can be expected. SNPs influencing RNA modification regulate susceptibility genes, thereby participating in the pathogenesis of CHD and DM2.^[49]^ These SNPs can facilitate the identification of regulatory enhancers in different cell types and, therefore, understand how SNPs lead to changes in gene expression regulation.

The endophenotype that links DM2 and CHD. Variations in 9p21 are currently the most reliable markers for cardiovascular disease outcomes in large-scale GWAS. In patients with a 9p21 risk genotype, fasting plasma insulin levels are higher compared to the non-risk group. Both DM2 and CHD are caused by impaired insulin signaling and subsequent insulin resistance. Insulin resistance is associated with atherosclerosis through the pro-inflammatory activity of immune and vascular cells, which are also regulated at the 9p21.3 locus.^[50]^ Even in the absence of hyperglycemia, insulin resistance contributes to early atherogenesis, endothelial dysfunction, and the development and progression of CHD. Both diseases, having a shared innate genetic risk at the level of cell cycle regulation, can develop independently based on their own metabolic phenotypes. Abdul-Ghani T et al identified 3 distinct metabolic phenotypes of DM2, characterized by different clusters of DM2 and differential responses to glucose-lowering therapy.^[51]^ Nair, et al concluded that different phenotypes of DM2 have distinct outcomes and responses to medication. For example, patients in the European population with retinopathy exhibit phenotypic differences compared to patients with CHD and also demonstrate a different treatment response.^[51]^

Personalized interventions targeting the underlying causes and progression, as well as intracellular biomolecular protein condensates, deserve attention as a unified approach in the concurrent onset of CHD and DM2. Many scientific studies emphasize that it is DM2 that causes CHD,^[51]^ although CHD may precede DM2. DM2 patients carrying the risk allele of the 9p21.3 locus more often experience severe CHD and poorly controlled hyperglycemia, which develops indirectly through vascular endothelium and pro-inflammatory processes.^[52,53]^ The Mendelian randomization study demonstrated that the association between DM2 and CHD risk is age-dependent at the onset of DM2.^[12]^

Thus, the assumptions about the shared starting point of DM2 and CHD development as a result of a transcriptional error at the 9p21.3 chromosomal locus influenced by ANRIL are quite convincing and continue to be supplemented through scientific research.^[54]^ DM2 and CHD represent a complex phenotype determined by genetic factors and environmental factors. However, by developing together, they represent a dangerous phenotypic endotype for the patient health and life, requiring early intervention. Understanding that disturbances in the genome sequence at the 9p21.3 chromosomal locus often contribute to their development can serve as a basis for such intervention.

Certainly, there are some limitations to the presented study. Firstly, the list of publications whose results were used in this review is not exhaustive. It is limited to the 9p21.3 chromosomal locus and the symptoms associated with DM2 and CHD risk, as well as the availability of publications to the review authors. Secondly, not all presented data have compelling numerical evidence of their existence, and decades of observation may be required for such evidence. Additionally, some cases show unsuccessful molecular analysis studies, which, on the one hand, illustrate the complexity of this interaction and, on the other hand, hold promising prospects for future research. However, despite the limitations, the review presents the most significant medically relevant sequential stages of the 9p21.3 locus investigation concerning the consensus genetic risk of DM2/CHD.

## 5. Conclusions

Access to more advanced technologies contributes to progress in acquiring new scientific knowledge about the prospects of diagnosing and treating chronic non-communicable diseases. Systematizing research ideas, from fragmented to systematic study of the overall development of DM2 and CHD in relation to the genetic locus 9p21.3, can serve as a source of new hypotheses for effective interventions in managing the growing global burden of DM2 and CHD. In the short term, the results of our review can be seen in the inclusion of genetic risk at the level of the chromosomal locus 9p21.3 in decision-making algorithms for patients with the phenotype of “DM2 + CHD.” Indeed, understanding the biological processes related to the chromosomal locus 9p21.3 gains practical significance, as for example, by focusing on the condensates and their associated proteins, researchers and biotech companies aim to develop novel approaches to mitigate the effects of CHD and T2D and improve patient outcomes.

## Acknowledgments

Special thanks to Valery Benberin (MD, MDSc) the head of the Center of Gerontology and Rauza Bigarinova (MD, MBA) for contributing in winning the funding for the research.

## Author contributions

**Conceptualization:** Rauza Bigarinova.

**Data curation:** Valeriy Benberin.

**Funding acquisition:** Valeriy Benberin.

**Investigation:** Nazgul Kulmyrzaeva.

**Methodology:** Raushan Karabaeva.

**Project administration:** Rauza Bigarinova, Valeriy Benberin.

**Resources:** Raushan Karabaeva.

**Validation:** Raushan Karabaeva.

**Writing – original draft:** Tamara Vochshenkova, Nazgul Kulmyrzaeva, Rauza Bigarinova.

**Writing – review & editing:** Tamara Vochshenkova.
